# Patient activation for self‐management among adult patients with multimorbidity in primary healthcare settings

**DOI:** 10.1002/hsr2.735

**Published:** 2022-07-20

**Authors:** Leila Paukkonen, Anne Oikarinen, Outi Kähkönen, Pirjo Kaakinen

**Affiliations:** ^1^ Research Unit of Nursing Science and Health Management University of Oulu Oulu Finland; ^2^ Medical Research Centre Oulu Finland

**Keywords:** chronic conditions, health‐related quality of life, multimorbidity, patient activation, primary healthcare, self‐management

## Abstract

**Background and Aims:**

Multimorbidity is a major public health and healthcare challenge around the world, including in Finland. As multimorbidity necessitates self‐management in everyday life, the effects of patient activation – a patient's knowledge, skills, and confidence in managing own health – on the capacity for self‐management warrant study, especially in primary healthcare settings. This study aimed to assess patient activation among multimorbid primary healthcare patients, identify factors associated with patient activation, and determine whether patients with low and high activation differ in terms of health and self‐management behavior, related perceptions, and health‐related quality of life (HRQoL).

**Methods:**

A cross‐sectional survey was conducted among multimorbid patients who attended Finnish primary healthcare consultations (November 2019 to May 2020). The main outcome, patient activation, was assessed using the patient activation measure, PAM‐13®. Responses from 122 patients were analyzed using descriptive statistics, *t*‐tests, analysis of variance, linear modeling, the *χ* ^2^ test, and binary regression analysis.

**Results:**

The mean score of patient activation was 56.12 (SD 12.82) on a scale 0–100 where ≤55.1 indicate low activation. The lower activation scores were significantly associated with old age, obesity, loneliness, and lower perceived health, functional ability, and vitality. Patients with low activation (47%) had significantly poorer physical activity, diets, adherence to care, and HRQoL, and significantly worse perceptions related to self‐management including motivation and energy, sense of normality, and support from physicians, nurses, and close people.

**Conclusion:**

Patient activation among multimorbid outpatients was rather low. Findings indicate that patients' perceptions of their health and psychosocial factors may be important for activation and that patients with low and high activation differ with respect to several health variables. Determining patient activation in multimorbid patients may facilitate adaptation of care to better meet patient capabilities and needs in clinical settings. Knowledge of a patient's activation level may also be useful when developing interventions and care strategies for this patient group.

## 1 INTRODUCTION

Multimorbidity, defined as the coexistence of two or more chronic conditions in the same individual, is increasingly common worldwide, and also in Finland.^1^, ^2^ The prevalence of multimorbidity varies according to the population and methods used to measure it, but approximately one‐fourth of the population, and over half of the population over 65 years of age, have multimorbidity.^3^, ^4^, ^5^, ^6^ The risk of multimorbidity increases with age, as does the number of related conditions.^4^, ^5^ Also, an unhealthy lifestyle^7^, ^8^, ^9^ and socioeconomic disadvantage^3^, ^4^, ^5^, ^8^ seem to be linked to an increased risk of developing multimorbidity. Multimorbidity is associated with many profound, and negative, outcomes such as decreased functional status, perceived health,^10^, ^11^, ^12^ and quality of life,^11^, ^12^ as well as increased loneliness,^13^, ^14^ treatment burden,^15^ and health service utilization.^10^, ^16^ Primary healthcare patients with multimorbidity represent a prominent part of the workload^11^, ^17^ more than half of the consultations^5^, ^11^ and the most of prescriptions.^5^ Multimorbidity also increases the likelihood of hospital admission, length of hospital stays and readmission, and overall healthcare utilization and costs.^16^

However, individuals with multimorbidity are in contact with their healthcare providers for only a fraction of their lives, while the most of time they are managing their condition on their own on a day‐to‐day basis. For example, they are responsible for adhering to prescribed medication regimes, self‐monitoring their condition, and maintaining a healthy diet and adequate physical activity.^18^, ^19^ Self‐management, defined as an “individual's ability to manage the symptoms, treatment, physical and psychosocial consequences, and lifestyle changes inherent in living with a chronic condition,”^20^ is crucial in the care of individuals with multimorbidity. Successful self‐management helps us to minimize troublesome symptoms, prevents the onset of additional illnesses, and allows patients to maximize their quality of life despite their chronic conditions.^19^ However, life‐long self‐management of even one chronic condition can be challenging, and self‐management can be complex and onerous for patients with multiple chronic conditions. This often necessitates coordination of care between different providers as well as management of complicated and demanding medical needs along with competing and potentially conflicting priorities and self‐management regimens.^18^, ^21^ The burden of treatment facing many multimorbid patients can thus be compared to the burden of diseases itself.^15^ As a such, success of self‐management is emphasized by one's ability and willingness to be involved in the care process, which is often referred to as patient activation. Specifically, patient activation entails having the knowledge and skills to manage one's own health and healthcare, and confidence in managing health‐related tasks.^22^, ^23^

According to the construct of patient activation more highly activated individuals believe that their role in managing their own health is important, have the knowledge and confidence necessary to act appropriately, and enact behaviors to maintain or improve their health.^22^, ^23^ Accordingly, previous empirical studies have found that patient activation is linked to many positive health behaviors among patients with diverse chronic conditions. More activated patients are more likely to engage in self‐management behaviors including physical exercise,^24^, ^25^, ^26^ healthy diet,^27^, ^28^ not smoking,^25^, ^29^ and following medication guidelines, and monitoring their condition.^30^, ^31^ Furthermore, studies have also found that higher activation is predictive of positive clinical outcomes including better blood glucose and pressure control^29^, ^32^ and lower body mass index (BMI),^25^, ^33^, ^34^ and that those with higher activation tend to have better perceived health^32^, ^35^ and health‐related quality of life (HRQoL).^36^, ^37^, ^38^ Additionally, high patient activation has been shown to be associated with lower healthcare utilization^29^, ^39^, ^40^, ^41^ and costs.^42^

Patient activation is also associated with varied psychological and psychosocial factors. Studies on patients with chronic conditions have shown that those with lower activation tend to experience depressive symptoms/depression^33^, ^36^, ^38^ and anxiety^34^, ^38^ more frequently and have lower satisfaction with perceived social role^38^ and social support.^33^, ^36^ Patient activation also seems to be linked to experiences of diverse positive and negative emotions and feelings related to illness^43^ and managing health.^44^ Hibbard and Mahoney^44^ found that those with low levels of activation feel more often overwhelmed and less motivated in managing their own health than those with high levels of activation. Furthermore, some studies have shown that good perceived quality of patient–physician relationship was associated with higher patient activation.^43^, ^45^ A recent systematic scoping review confirmed that psychosocial and psychological factors seem to explain variations in patient activation, but that the role of these factors in influencing patient activation has so far little been studied.^46^

Thus, previous studies in patients with a chronic condition(s) have shown the proven beneficial impact of patient activation on self‐management and found several patient‐related factors associated with patient activation. However, despite the need for more research on improving the care of patients with multimorbidity, a priority for global health research^47^ only a few studies have investigated patient activation for self‐management in this population. Thus, there are clear needs for research on the subject. Knowledge of patient activation levels and factors associated with patient activation among multimorbid primary care patients could facilitate the identification of patients who need more support in self‐management and tailoring of counseling and care to meet patients' needs and capabilities, while also revealing potential risk factors for low activation. This in turn would inform interventions needed for this patient group.

Hence, the study's aim is to assess patient activation among multimorbid primary healthcare patients and identify factors related to patient activation in this population. Factors included were: sociodemographic (age, gender, education level, employment status, marital status, and living situation) and health‐related and psychosocial factors (number of conditions, obesity, perceived health, perceived functional ability, perceived vitality, and perceived loneliness). In addition, the study aims to determine whether patients with low and high activation differ in terms of self‐management behavior (physical activity, diet, use of alcohol and tobacco products; and adherence to care regimens) and perceptions related to self‐management, as well as HRQoL.

To this end, three research questions were posed:

1. What is the patient activation status of patients with multimorbidity in this study?
2. How are patient activation scores associated with sociodemographic, health‐related, and psychosocial factors?
3. How do patients with low and high levels of activation differ in terms of self‐management behavior, related perceptions, and HRQoL?

## 2 METHODS

### 2.1 Study design, eligible participants, and data collection

This study used a cross‐sectional research design with survey data. Participants were adult outpatients with multimorbidity who attended Finnish primary healthcare consultations for chronic condition management. The survey was conducted among those patients who attended consultations during the data collection period (November 2019 to May 2020) in one municipality in which all health centers participated in the study. Recruitment was performed by health personnel, mainly nurses who were responsible for the patients' chronic care, and took place in‐person appointments with a nurse or a doctor. Before data collection, service managers at the participating health centers were briefed on the study and subsequently distributed information about the study to their staff. Personnel were instructed to distribute questionnaires to all patients satisfying the eligibility criteria. The main inclusion criterion of the study was multimorbidity; the coexistence of two or more chronic conditions, all of which were either: A physical noncommunicable disease of long duration (e.g., cardiovascular disease, diabetes, asthma, or cancer), a mental health condition of long duration (e.g., a mood disorder), and an infectious disease of long duration such as HIV or hepatitis C.^47^ Participants were also required to be at least 18 years old and to be sufficiently proficient in Finnish to complete a questionnaire. Questionnaires included detailed written information about the study purpose and objectives, the researchers' contact information, as well as a return postal envelope.

### 2.2 Study measures and variables

The main outcome variable was patient activation, which was assessed using the patient activation measure, PAM‐13®. PAM is widely used and validated in different patient populations.^22^, ^23^, ^48^ PAM generates scores of 0–100, with higher scores indicating greater patient activation. Based on their scores, respondents can be divided into four developmental activation levels ranging from a passive recipient to an active manager of their own health. Both scores and levels are usable^22^, ^23^, ^48^ (for more details, see Tables 1 and 3). The present study used the Finnish‐language version of PAM, which is available under license from Insignia Health. The Cronbach's *α* value for PAM was 0.82 in this study.

**Table 1:** Information collected and measurements used in the study

*Patient activation*	
Patient Activation Measure® (PAM‐13)®	*PAM* is unidimensional measure containing 13 items measuring patient activation: Self‐assessed knowledge, skills, and confidence in self‐management of chronic condition(s) and health.^22^, ^23^ *Answerin*g: Items are answered using 4‐point Likert scale (1–4) ranging from disagree strongly to agree strongly, and an additional “not applicable” option (no score, the data is treated as missing). *Scoring*: The raw scores are summed (range 13–52) and then converted into continuous patient activation scale scores (using a calibration table provided by Insignia Health) between 0 and 100 where higher scores indicate greater activation. These scores can be stratified into four activation levels, with Level 1 being the least activated and Level 4 the most activated, correspond to scores of <47.1, 47.1 to 55.1, 55.2–67.0, and >67.1, respectively. These levels can also be used as cutoffs.^48^ In this study Cronbach's *α* was 0.82
*Sociodemographic information*	
Demographic information	Age, gender, education level, employment status, marital status, and living situation (alone with partner, etc.)
*Health‐related and psychosocial information*	
Chronic conditions constituting multimorbidity	The form included 26 conditions listed: diabetes, hypertension, coronary heart disease, heart failure, arrhythmia, stroke, rheumatoid arthritis, ankylosing spondylitis, osteoarthritis, cancer, chronic obstructive pulmonary disease, asthma, hypo‐ or hyperthyroidism, inflammatory bowel disease (Crohn's Disease, ulcerative colitis, etc.), chronic kidney disease, memory disorder (like Alzheimer's disease), allergy, celiac disease, depression, bipolar disorder, schizophrenia, Parkinson's disease, epilepsy, multiple sclerosis, glaucoma, and hepatitis C. *Answering*: Respondents could select any combination of diagnosed chronic conditions from the list and add other long‐term diseases not mentioned in the list in a text box.
Height and weight	Height and weight were used to calculate a body mass index (BMI) by researchers. *BMI scoring*: Body mass in kilograms divided by height in meters squared (BMI = kg/m^2^). Obesity is defined as a BMI of 30 kg/m^2^ or more; overweight as a BMI of 25–29.9 kg/m^2^; normal weight as BMI of 24.9 to 18.5 kg/m^2^; and underweight as a BMI below 18.5 kg/m^2^.^49^
Perceived health	A subjective rating by the respondent of his or her general health status. Universally widely used one question indicator.^50^ *Answering*: Possible answer options were good, quite good, fair, quite poor, and poor; consistently with the study of the Finnish Institute for Health and Welfare.^51^
Perceived functional ability	A subjective rating of the respondent's experience of his/her ability to cope with meaningful and necessary daily life activities in the environment in which they live. One question. *Answering*: Possible answer options were good, quite good, fair, quite poor, and poor.^52^
Perceived loneliness	Single question with answer options not at all, sometimes, and often
Perceived sufficient number of close friends and relatives	Single question with answer options sufficiently, not sufficiently, not at all
Health‐related quality of life (HRQoL)	*15D* is a generic instrument for measuring HRQoL the people's assessment of their health‐related well‐being, including 15 dimensions: mobility, vision, hearing, sleeping, eating, speech excretion, usual activities, mental function, discomfort and symptoms, depression, distress, vitality, and sexual activity.^53^ *Answering*: Items are answered using 5‐point ordinal scales. Respondent chooses from each dimension the level, which best describes her/his present health status. *Scoring*: Instrument combines a profile (a 15‐dimensional description of persons health status) and a preference‐based, single index measure. The scoring algorithm is provided by the meter administrator.^54^ In this study, Cronbach's *α* was 0.87
*Self‐management behavior*	
Physical activity	*The FIT index of Kasari* developed by Kasari in 1976 is based on of three parameters: frequency of exercise (how often do you exercise), intensity of exercise (with what intensity do you usually exercise), and time spent on workout (how long do you usually work out). Each parameter has one corresponding question. FIT scores can be used to assess general physical activity.^55^ *Answering*: The frequency and intensity questions are answered on 5‐point scales. For frequency, 1 represents once per month or less and 5 represents at least six times per week. For intensity, 1 represents light aerobic activity and 5 represents high intensity. The time question is answered on a 4‐point scale where 1 represents less than 10 min and 4 represents >30 min. *Scoring*: The FIT index is calculated according to participants' responses by multiplying the scores obtained for each parameter as follows: FIT index = (points for frequency) × (points for intensity) × (points for duration). FIT scores range from 1 (low activity) to 100 (high activity), with scores of <36, 37–63, and > 64 indicating low, moderate, and high physical activity levels, respectively.^56^ In this study, Cronbach's *α* was 0.78
Alcohol consumption	*Alcohol use disorders identification test (*AUDIT*)‐consumption* is the first 3 questions of 10 question the AUDIT‐instrument developed by the World Health Organization.^57^ *Answering*: Each question has five possible answers. *Scoring*: Each answer is assigned between 0 and 4 points, and the points are summed. On a scale ranging from 0–12, scores of 0 reflect no alcohol use. Scores of 4 or more in men and 3 or more in women are considered positive. Points below these numbers indicate low risk, scores of up to 5 indicate moderate risk, and scores of 6 or more indicate high risk in both genders.^58^ In this study Cronbach's *α* was 0.91
Tobacco use	Two questions: “At the present time, do you smoke cigarettes/do you use other tobacco products (snuff, chewing tobacco etc)?” Answer options: Not at all, occasionally, daily.
Diet	Two single items: *Assess the variety of your diet*. Answering: by 5‐point Likert scale from very good to very one sided. *Do you follow the dietary according to the agreed instructions?* Answering: by 4‐point Likert scale (1–4) with options: totally disagree, partly disagree, partly agree, totally agree
Adherence to chronic care	Adherence of people with chronic disease instrument (ACDI), originally developed by Kyngäs^59^ is validated for use with patients with various chronic diseases. It contains 11 statements concerning agreed chronic care including medications, general care regimens, diet, monitoring, co‐operation, responsibility, and willingness to own care. *Answering*: Items are answered using 4‐point Likert scale (1–4) ranging from totally disagree to totally agree. *Scoring*: Mean sum variable 1–4, with scores of ≥3.5 indicating good value. In this study, Cronbach's *α* was 0.77
*Self‐management perceptions*	
	26 statements on *ACDI‐scale* ^59^ as follows: Energy and motivation (two items) Cronbach's *α* was 0.76 Sense of normality (nine items): Cronbach's *α* was 0.83 Fear of complications and additional diseases (two items) Cronbach's *α* was 0.94 Support from physicians (four items) Cronbach's *α* was 0.86 Support from nurses (four items) Cronbach's *α* was 0.89 Support from family and friends (five items) Cronbach's *α* was 0.80 *Answering*: Items are answered using 4‐point Likert scales (1–4) with options totally disagree, partly disagree, partly agree, totally agree *Scoring*: Mean sum variables 1–4, with scores of ≥3.5 indicating good value

Other variables considered include (I) sociodemographic factors: age, gender, education level, employment status, marital status, and living situation, (II) health‐related and psychosocial factors: chronic conditions, height, and weight to calculate BMI,^49^ perceived health,^50^, ^51^ perceived functional ability,^52^ HRQoL evaluated using the 15D instrument,^53^, ^54^ and perceived loneliness and a sufficient number of close friends and relatives, (III) self‐management behavior: physical activity assessed using the frequency intensity time (FIT) index of Kasari,^55^, ^56^ alcohol consumption assessed using the alcohol use disorders identification test‐consumption,^57^, ^58^ tobacco consumption and diets both evaluated using two single questions, and adherence to care regimens, and (IV) perceptions related self‐management assessed using the adherence of people with chronic disease instrument.^59^ All used instruments were previously generally widely used validated tools and exhibited good internal consistency in this study. For further details, see Table 1.

### 2.3 Statistical analyses

All statistical analysis was performed using IBM SPSS for Windows (version 27.0; IBM Corporation). Descriptive statistics: means, standard deviations (SDs), and ranges were used to describe continuous variables, while frequencies, percentages, and distributions were used for categorical variables. Evaluations of normality and outliers were performed using tests of normality and by visual inspection of data (creating histograms and boxplots). Data obtained from the questionnaires were also classified for some analyses. For all analyses, *p *< 0.05 was considered significant.

PAM scores for the study sample were obtained from Insignia Health. According to their guidelines, respondents must answer 10–13 questions (not applicable [N/A] responses are considered missing) to obtain a valid PAM score. Because PAM is a Guttmann‐like scale characterized by increasing difficulty as the survey progresses, uniform response patterns without variation are often considered unreliable and invalid. The sample consisted of 122 patients. Questionnaires with more than three N/A or missing responses (*n* = 16) were deleted to ensure validity, as were variables those for which all questions were answered using the same response option (*n* = 6). All subsequent analyses were based solely on the non‐excluded questionnaires (*n* = 100; see Table 2). The sample size for this study was thus determined by availability of responses; however, a power analysis regarding PAM measurement using the final sample size of 100 at *α* = 0.05 showed the achieved power was 0.87 for the *χ* ^2^ test and 0.95 for analysis of variance (ANOVA; for perceived functional ability and perceived health) and thus well above the threshold of 0.80.

**Table 2:** Sociodemographic characteristics of the study participants with multimorbidity (*n*, %)

Characteristics	*n* (122)	Sample with PAM score (*n* = 100)
	*n* (%)	*n* (%)
*Age*		
38–64 year	39 (32.0)	32 (32.0)
65–74 years	47 (38.5)	41 (41.0)
75–93 years	34 (27.9)	25 (25.0)
Missing	2 (1.6)	2 (2.0)
*Gender*		
Female	71 (58.2)	58 (58.0)
Male	51 (41.8)	42 (42.0)
*Education*		
Primary	35 (28.7)	29 (29.0)
Secondary (high school/vocational education)	25 (20.5)	19 (19.0)
Tertiary	62 (50.8)	52 (52.0)
*Employment status*		
Employed	11 (9.0)	10 (10.0)
Unemployed or long‐term sick leave	5 (4.1)	4 (4.0)
Retired	106 (86.9)	86 (86.0)
*Marital status*		
Single	13 (10.7)	12 (12.0)
Married/In a registered partnership	78 (63.9)	61 (61.0)
Divorced	20 (16.4)	19 (19.0)
Widowed	11 (9.0)	8 (8.0)
*Living situation*		
Alone	35 (28.7)	30 (30.0)
With spouse/partner	63 (51.6)	52 (52.0)
With spouse/partner and child/children	21 (17.2)	15 (15.0)
With someone other	3 (2.5)	3 (3.0)

Abbreviation: PAM, patient activation measure.

Patient activation was evaluated as both a continuous variable (PAM scores) and a categorical variable; the four activation levels (1–4) originally suggested were dichotomized into low activation (Levels 1 and 2) and high activation (Levels 3 and 4), in accordance with previous studies.^60^, ^61^ First, the associations of continuous PAM scores and categorial patient characteristics were assessed; More specially, the statistical significance of differences in mean PAM between groups was evaluated using independent samples *t*‐tests for dichotomous categorical patient variables and one‐way ANOVA for variables with more than two categories, with the Tukey test for post hoc comparisons. Second, differences between those with low and high activation in terms of self‐management behavior and related perceptions were explored. When comparing means for low and high activation groups the independent samples *t*‐tests were used. In addition, because PAM scores were previously found to be associated with patient‐related factors, the association of patient activation level with self‐management behaviors and perceptions was also calculated by adapting perceived health, loneliness, and obesity in a linear model. Also, because HRQoL is known to be related to age, gender, and disease count,^62^ the association of activation level with HRQoL dimensions was also calculated by adjusting these factors in the linear model. Differences between proportions for categorical variables were compared using the *χ* ^2^ test. Further, binary logistic regression analysis with the calculation of odds ratios (ORs) was used to identify effects between patients' activation (low and high) and self‐management behaviors and perceptions.

## 3 RESULTS

### 3.1 Sample characteristics

The mean age of the participants was 68 years (SD 11.4). Over half (58%) of the respondents were women; 42% were men. Somewhat less than a third (29%) had only a basic education, one‐fifth (19%) had completed secondary education, and a half (52%) had completed at least postsecondary education. The majority (86%) of the participants were retired. Well over half were married or in a registered partnership (61%) and half lived with a spouse or partner (52%). About a third (30%) lived alone (Table 2).

Participants had on average four chronic conditions (range: 2–13), and a wide variety of conditions and diseases were represented. The most common chronic physical conditions were hypertension (suffered by 73% of participants), diabetes (62%), asthma (28%), coronary artery disease (28%), arrhythmia (23%), and osteoarthritis (23%). Twelve percent of participants suffered from depression. Additionally, 46% of the participants were obese, 36% were overweight, and 18% were of normal weight.

### 3.2 Patient activation status

The mean PAM score was 56.12 (SD = 12.82; 95% confidence interval = 53.65–58.58) based on a theoretical point scale ranging from 0 to 100. The distribution of PAM levels was as follows: 23% of participants were at Level 1 (“disengaged and overwhelmed”), 24% were at Level 2 (“becoming aware, but still struggling”), 46% were at Level 3 (“taking action”), and 7% were at Level 4 (“maintaining behaviors and pushing further”). Thus, 47% of participants exhibited low activation (Levels 1 and 2) and 53% exhibited high activation (Levels 3 and 4). For further details, see Table 3.

**Table 3:** The levels of patient activation^48^ and their proportion in this study

PAM level		PAM score (possible range 0–100)	Interpretation	Proportion in this study
			*“Disengaged and overwhelmed”*	23%
Low	Level 1	<47.0	Individuals tend to be passive and lack knowledge and confidence. They may not yet understand their role in care process and managing their health.	
			*“Becoming aware, but still struggling”*	24%
	Level 2	≥47.1 and ≤55.1	Individuals have some knowledge, but large gaps remain, and they still lack the confidence to manage their health. They may believe health is largely out of their control.	
			*“Taking action”*	46%
High	Level 3	≥55.2 and ≤67.0	Individuals appear to be taking action and building self‐ management skills but may still lack the confidence and skill to maintain their behavior.	
			*“Maintaining behaviors and pushing further”*	7%
	Level 4	≥67.1	Individuals have adopted many of the behaviors needed to support their health but may not be able to maintain them in the face of stress or change.	

Abbreviation: PAM, patient activation measure, PAM‐13®.

### 3.3 Factors associated with patient activation score

The only sociodemographic factor significantly associated with the PAM score was age, although this association appeared only when comparing the oldest participant group (75 years or older) to the others (*M*= 51.75; *M* = 58.20, *p* = 0.035). The patient's number of chronic conditions was not related to patient activation. However, an association was found for obesity: nonobese participants had higher PAM scores (*M* = 60.04) than obese participants (*M* = 54.63, *p* = 0.032). Additionally, higher PAM scores were associated with better perceived health (*p* = 0.019), functional ability (*p* = 0.016), and vitality (*p* = 0.001). There were also statistically significant differences in PAM score based on the experience of loneliness: patients with perceived sufficient number of close friends and relatives had higher PAM scores (*M* = 58.14) than those without sufficient close friends and relatives (*M* = 51.78, *p* = 0.031). Finally, participants who felt lonely reported lower activation (*M* = 52.99) than those who did not (*M* = 58.91, *p* = 0.025) (Table 4).

**Table 4:** Sample characteristics and their associations with the PAM score

Characteristics	*n* (%)	Mean (SD)	*p*‐Value^a^	Mean difference (95% CI)^b^
*Age*				
≤64 year	32 (32.0)	57.37 (13.47)	0.097	Ref.
65–74 years	41 (41.0)	58.84 (11.89)		−8.80 to 5.85
≥75 years	25 (25.0)	51.75 (14.28)		−2.67 to 13.91
Missing	2 (2.0)			
<74 years	73 (74.5)	58.20 (12.54)	**0.035**	0.47–12.43
≥75 years	25 (25.5)	51.75 (14.27)		
*Gender*				
Female	58 (58.0)	56.88 (14.52)	0.688	−4.23 to 6.38
Male	42 (42.0)	55.80 (11.09)		
*Education*				
Primary	29 (29.0)	54.39 (10.59)	0.585	Ref.
Secondary (high school/vocational education)	19 (19.0)	56.40 (12.35)		−11.28 to 7.26
Tertiary	52 (52,0)	57.57 (14.69)		−10.46 to 4.11
*Employment status*				
Employed	10 (10.0)	60.38 (14.50)	0.381	Ref.
Unemployed or long‐term sick leave	4 (4.0)	49.75 (10.25)		−7.88 to 29.14
Retired	86 (86.0)	56.27 (13.08)		−6.34 to 4.56
*Marital status*				
Single	12 (12.0)	50.97 (13.07)	0.297	Ref.
Married/In a registered partnership	61 (61.0)	58.25 (13.86)		−18.09 to 3.53
Divorced	19 (19.0)	54.52 (10.98)		−16.17 to 9.07
Widowed	8 (8.0)	55.16 (11.20)		−19.81 to 11.43
*Living situation*				
Alone	30 (30.0)	54.75 (11.66)	0.665	Ref.
With spouse/partner	52 (52.0)	57.58 (12.55)		−10.77 to 5.10
With spouse/partner and chid/children	15 (15.0)	57.00 (17.30)		−13.20 to 8.69
With someone other	3 (3.0)	50.17 (18.06)		−16.37 to 25.53
*Perceived loneliness*				
Not at all	58 (58.0)	58.91 (13.66)	**0.025**	0.751–11.10
Yes	42 (42.0)	52.99 (11.69)		
*Perceived sufficient number of close friends and relatives*				
Yes, sufficiently	73 (73.0)	58.14 (13.01)	**0.031**	0.618–12.11
No, not sufficiently/not at all	27 (27.0)	51.78 (12.57)		
*Number of chronic conditions*				
2–3	48 (49.5)	56.30 (12.86)	0.783	Ref.
4–5	27 (27.8)	57.36 (11.77)		−8.63 to 6.50
6–13	22 (22.7)	54.71 (15.43)		−6.50 to 9.68
*Obesity*				
<BMI 30	51 (53.7)	60.04 (13.06)	**0.032**	0.48–10.34
BMI ≥30.0	44 (46.3)	54.63 (10.78)		
*Perceived health*				
Good or quite good	50 (50)	59.54 (10.80)	**0.019**	Ref.
Fair	32 (32.3)	55.58 (14.89)		−3.14 to 10.52
Quite poor or poor	17 (17.2)	49.40 (13.30)		1.67–18.61
*Perceived functional ability*				
Good or quite good	58 (58.6)	59.73 (10.65)	**0.016**	Ref.
Fair	21 (21.2)	53.03 (17.11)		−0.97 to 14.37
Quite poor or poor	20 (20.2)	51.32 (12. 66)		0.60–16.22
*Vitality*				
Good	29 (29.00)	63.27 (13.48)	**0.001**	4.20–15.08
Not good (=weary, tired, or feeble)	71 (71.00)	53.63 (12.00)		

*Note*: Statistical significance recognized as *p* < 0.050. Bold values are statistically significant.

Abbreviations: ANOVA, analysis of variance; BMI, body mass index; CI, confidence interval; PAM, patient activation measure.

^a^ *t*‐test for pairwise comparisons and one‐way ANOVA for three or more groups.

^b^ Post hoc comparison with Tukey method.

### 3.4 Comparison between low and high activation level patients

Patients with low patient activation had significantly lower physical activity based on the FIT index than those with high activation (*M* = 27.8; *M* = 39.6, *p* = 0.002, *t*‐test; the result also remained significant (*p* = 0.015) even when adjusted with obesity, loneliness, and perceived health in same model) and more frequently had sedentary lifestyles or only some physical activity (73.8%) than those with high activation (45.3%, *p* = 0.004, *χ* ^2^). Patients with low activation were also more likely to eat one‐sided manner (55.3%) than those with high activation (24.5%, *p* = 0.002, *χ* ^2^), and followed agreed dietary instructions less frequently than those with high activation (*p* = 0.007, *χ* ^2^). Furthermore, there was a significant difference in the odds of following a moderate‐high physical activity (OR: 3.41, *p* = 0.006), a varied diet (OR: 3.81, *p* = 0.002) and a diet according to agreed instructions (OR: 3.33, *p* = 0.008) between patients with high activation compared to patients with low activation. Finally, the low activation group reported lower adherence to care regimens than the high activation group (*M* = 3.4; *M* = 3.7, *p* = 0.001, *t*‐test). This result was revealed to be significant even when adjusted for obesity, loneliness, and perceived health in the same model (*p* = 0.006). Furthermore, there was a significant difference in the odds of good adherence between high‐ and low‐activation patients (OR: 3.82, *p* = 0.006) (Table 5).

**Table 5:** Differences in self‐management behaviors between participants at different levels of patient activation (mean [SD] or *n* (%), *p*‐values, and effect size) and whether activation level is an explanatory factor for behaviors (odds ratio [OR], 95% confidence interval [CI], and *p*‐value)

	High patient activation	Low patient activation			High versus low patient activation^a^	
Factor	*n* (%) or mean (SD)	*n* (%) or mean (SD)	*p*‐Value effect size	*p*‐Value adjusted^b^	OR (95% CI)	*p*‐Value
*Alcohol use*						
No risky use	40 (78.4)	40 (87.0)	0.270^c^		0.55 (0.18–1.62)	0.274
Yes, risky use	11 (21.6)	6 (13.0)	0.112^d^			
*Tobacco use*						
No	46 (86.8)	41(87.2)	0.948^c^		0.962 (0.30–3.10)	0.948
Yes	7 (13.2)	6 (12.8)	0.007^d^			
*Physical activity*						
Moerate or High	29 (54.7)	11 (26.2)	**0.005** c		3.41 (1.40–8.17)	**0.006**
Low or sedentary	24 (45.3)	31 (73.8)	0.287^d^			
FIT‐index score	39.6 (18.7)	27.8 (18.0)	**0.002** e	**.015** b		
			0.642^f^			
*Diet*						
Yes, varied diet	40 (75.5)	21 (44.7)	**0.002** c		3.81(1.63–8.91)	**0.002**
Not varied diet	13 (24.5)	26 (55.3)	0.315^d^			
Totally agree	23 (43.4)	8 (17.8)	**0.007** c		3.33 (1.39–9.06)	**0.008**
Others	30 (56.6)	37 (82.2)	0.275^d^			
*Adherence to overall chronic care regimens*						
Good	45 (84.9)	28 (59.6)	**0.004** c		3.82 (1.47–9.88)	**0.006**
Not good	8 (15.1)	19 (40.4)	0.285^d^			
ACDI score	3.7 (0.3)	3.4 (0.4)	**0.001** e	**0.006** b		
			0.735^f^			

*Note*: Statistical significance recognized as *p* < 0.050. Bold are statistically significant.

Abbreviations: ACDI, adherence of people with chronic disease instrument; SD, standard deviation.

^a^ Binary regression analysis.

^b^ Adjusted with obesity, loneliness, and perceived health.

^c^ Used test is *χ* ^2^ test.

^d^ Used test is Cramer's *V* with interpretation: 0.10 small, 0.30 medium, and 0.50 large.

^e^ Used test is independent samples *t*‐tests.

^f^ Used test is Cohen's *d* with interpretation: 0.2 small, 0.50 medium, and 0.80 large.

The low and high activation groups differed significantly on several factors relating to perceptions of self‐management: the high activation group had more positive perceptions in terms of having energy and motivation (*p* = 0.001, *t*‐test), feeling a sense of normality (*p* ≤ 0.001, *t*‐test), and having less fear of complications and additional diseases (*p* = 0.029, *t*‐test). The latter of which, however, was no longer statistically significant when adjusted for perceived health, loneliness, and obesity. Further, patients with low activation felt less support from physicians and nurses than high activated patients (*p* = 0.001 and *p* ≤ 0.0001, *t*‐test, respectively). Further, there was a significant difference in the odds of good energy and motivation (OR: 5.39, *p* < 0.001), good sense of normality (OR: 3.50, *p* = 0.004), and good support from physicians (OR: 5.46, *p* < 0.001), from nurses (OR: 6.40, *p* < 0.0001) and from family and friends (OR: 2.62, *p* = 0.020) between high‐ and low‐activation patients (Table 6).

**Table 6:** Differences in self‐management perceptions at different level of patient activation (mean [SD], *p*‐values, and effect size) and whether patient activation is explanatory factor for perceptions (odds ratio [OR], 95% confidence interval [CI], and *p*‐value)

	High patient activation	Low patient activation			High versus low patient activation a	
Factor	Mean (SD)	Mean (SD)	*p*‐Value^b^ effect size^c^	*p*‐value adjusted d	OR (95% CI)	*p‐*Value
Having energy and motivation	3.7 (0.7)	3.1 (0.8)	**0.001**	**0.028**	5.39 (2.10–13.87)	**<0.001**
			0.674			
Feeling sense of normality in own care	3.7 (0.5)	3.3 (0.5)	**<0.001**	**0.004**	3.50 (1.50–8.17)	**0.004**
			0.724			
Having no fear of complications and additional diseases	2.7 (1.0)	3.1 (0.8)	**0.029**	0.340	e	e
			0.443			
Receiving support from physicians	3.3 (0.6)	2.9 (0.6)	**0.001**	**0.006**	5.46 (2.15–13.87)	**<0.001**
			0.687			
Receiving support from nurses	3.6 (0.5)	3.1(0.6)	**<0.0001**	**<0.001**	6.40 (2.67– 15.35)	**<0.0001**
			0.875			
Receiving support from family and friends	3.5 (0.6)	3.3 (0.7)	0.091	0.103	2.62 (1.16–5.92)	**0.020**
			0.342			

*Note*: Statistical significance recognized as *p* < 0.050. Bold are statistically significant.

Abbreviation: SD, standard deviation.

^a^ Binary regression analysis with the dichotomous variable of self‐management perceptions: good (mean ≥ 3.5) versus others.

^b^ Used test is independent samples *t*‐tests.

^c^ Used test is Cohen's *d* with interpretation: 0.2 small, 0.50 medium, and 0.80 large.

^d^ Adjusted with obesity, loneliness, and perceived health.

^e^ Not calculated as only 2% of respondents reached the good value.

HRQoL also differed significantly between the patient activation groups; more specifically, patients with low activation had a significantly worse HRQoL (*p* = 0.001). HRQoL dimensions with statistically significant between‐group difference were breathing (*p* = 0.033), speech (*p* = 0.035), usual activities (*p* = 0.011), mental function (*p* = 0.014), depression (*p* = 0.004), distress (*p* = 0.025), vitality (*p* = 0.001), and sexual activity (*p* = 0.003). These differences remained statistically significant after adjusting for age, gender, and number of conditions. After adjusting for loneliness and obesity statistically significant between‐group differences were HRQoL total (*p* = 0.015), mental function (*p* = 0.021), depression (*p* = 0.014), distress (*p* = 0.042), vitality (*p* = 0.003), and sexual activity (*p* = 0.004) (Table 7).

**Table 7:** Health‐related quality of life (HrQoL) with 15 dimensions for participants with low and high activation, with and without adjustments for age, gender, number of conditions; and obesity, loneliness

Outcome					Linear model adjusted with age, gender, and number of conditions				Linear model adjusted with loneliness and obesity			
	Patient activation high	Patient activation low			Patient activation high	Patient activation low			Patient activation high	Patient activation low		
	Mean (SD)	Mean (SD)	Mean difference (95% CI)	*p*‐Value	Adjusted mean (SE)	Adjusted mean (SE)	Adjusted mean difference (95% CI)	*p*‐Value	Adjusted mean (SE)^†^	Adjusted mean (SE)	Adjusted mean difference (95% CI)	*p*‐Value
HrQoL (total)	0.88 (0.11)	0.80 (0.13)	0.08 (0.03–0.13)	**0.001**	0.88 (0.01)	0.80 (0.02)	0.08 (0.04– 0.12)	**0.000**	0.87 (0.02)	0.81 (0.02)	0.06 (0.01–00.10)	**0.015**
Mobility	0.87 (0.18)	0.80 (0.20)	0.07 (−0.01 to 0.14)	0.071	0.86 (0.02)	0.80 (0.03)	0.06 (−0.00 to 0.13)	0.061	0.85 (0.03)	0.83 (0.03)	0.02 (−0.05 to 0.10)	0.496
Vision	0.93 (0.13)	0.87 (0.20)	0.06 (−0.01 to 0.13)	0.081	0.93 (0.02)	0.87 (0.03)	0.06 (−0.00 to 0.13)	0.062	0.91 (0.02)	0.88 (0.02)	0.04 (−0.02 to 0.11)	0.201
Hearing	0.96 (0.11)	0.91 (0.17)	0.05 (−0.01 to 0.11)	0.100	0.95 (0.02)	0.91 (0.02)	0.04 (−0.02 to 0.10)	0.167	0.96 (0.02)	0.93 (0.02)	0.03 (−0.02 to 0.08)	0.265
Breathing	0.86 (0.23)	0.75 (0.26)	0.11 (0.01–0.20)	**0.033**	0.85 (0.03)	0.75 (0.03)	0.10 (0.01– 0.18)	**0.027**	0.85 (0.03)	0.79 (0.04)	0.06 (−0.04 to 0.16)	0.235
Sleeping	0.76 (0.23)	0.70 (0.23)	0.06 (−0.03 to 0.15)	0.179	0.77 (0.03)	0.72 (0.03)	0.05 (−0.04 to 0.13)	0.254	0.75 (0.03)	0.72 (0.04)	0.03 (−0.06 to 0.13)	0.501
Eating	0.99 (0.05)	0.98 (0.20)	0.01 (−0.02 to 0.04)	0.390	0.99 (0.01)	0.98 (0.01)	0.01 (−0.02 to 0.05)	0.430	0.99 (0.01)	0.99 (0.01)	0.00 (−0.02 to 0.02)	0.904
Speech	0.99 (0.06)	0.95 (0.11)	0.04 (0.00–0.07)	**0.035**	0.99 (0.01)	0.95 (0.01)	0.04 (0.00– 0.08)	**0.033**	0.99 (0.01)	0.97 (0.01)	0.03 (−0.01 to 0.06)	0.100
Excretion	0.86 (0.20)	0.78 (0.25)	0.08 (−0.01 to 0.17)	0.078	0.86 (0.03)	0.78 (0.03)	0.08 (−0.01 to 0.17)	0.090	0.85 (0.03)	0.79 (0.03)	0.06 (−0.04 to 0.15)	0.220
Usual activities	0.87 (0.19)	0.75 (0.26)	0.12 (0.03–0.21)	**0.011**	0.87 (0.03)	0.75 (03)	0.12 (−0.30 to 0.20)	**0.009**	0.85 (0.03)	0.79 (0.03)	0.06 (−0.02 to 0.14)	0.163
Mental function	0.91 (0.16)	0.81 (0.23)	0.10 (0.02–0.18)	**0.014**	0.91 (0.03)	0.82 (0.03)	0.09 (0.01–0.17)	**0.027**	0.91 (0.03)	0.81 (0.03)	0.10 (0.02–0.18)	**0.021**
Discomfort and symptoms	0.71 (0.27)	0.66 (0.24)	0.06 (−0.05 to 0.16)	0.280	0.71 (0.03)	0.64 (0.04)	0.07 (−0.02 to 0.17)	0.140	0.70 (0.04)	0.65 (0.04)	0.05 (−0.06 to 0.16)	0.393
Depression	0.91 (0.14)	0.81 (0.19)	0.10 (0.03–0.17)	0.**004**	0.91 (0.02)	0.82 (0.03)	0.09 (0.03–0.16)	**0.007**	0.90 (0.02)	0.81 (0.03)	0.09 (0.02–0.16)	**0.014**
Distress	0.89 (0.14)	0.82 (0.18)	0.07 (0.01–0.14)	**0.025**	0.90 (0.02)	0.83 (0.02)	0.07 (0.00–0.13)	**0.037**	0.89 (0.02)	0.82 (0.03)	0.07 (0.00–0.14)	**0.042**
Vitality	0.83 (0.19)	0.70 (0.18)	0.13 (0.06–0.20)	**0.001**	0.83 (0.02)	0.70 (0.03)	0.13 (0.07–0.20)	**0.000**	0.81 (0.03)	0.70 (0.03)	0.11 (0.04–0.19)	**0.003**
Sexual activity	0.78 (0.28)	0.61 (0.28)	0.17 (0.06–0.29)	**0.003**	0.74 (0.04)	0.60 (0.04)	0.14 (0.04–0.25)	**0.006**	0.77 (0.04)	0.60 (0.04)	0.17 (0.06–0.29)	**0.004**

*Note*: Statistical significance recognized as *p* < 0.050. Bold are statistically significant.

Abbreviations: CI, confidence interval; SD, standard deviation; SE, standard error.

## 4 DISCUSSION

This study provided new knowledge about patient activation and factors associated with patient activation for self‐management in patients with multimorbidity in Finnish primary healthcare settings, and differences related to self‐management between patients with low and high activation.

The mean activation score for the studied population was 56.1, which was quite low. According to a recent systematic review of patient activation in people living with chronic conditions, the mean PAM score in studies ranged from 59.1 to 82.5 (including 32 articles published between 2005 and 2019).^63^ However, the mean PAM score of this study is consistent with a previously reported value for multimorbid older adults.^26^ Nevertheless, the proportion of participants exhibiting the highest level of activation (4) was very low (7%). This is consistent with a study in patients with one or more selected chronic conditions that found that only a minority of patients scored PAM Level 4.^33^ Similar results were also reported previously for older patients with complex medical needs^35^ and frail older adults.^37^ This is noteworthy, among other things, in terms of health care resources, because in patients with chronic conditions activation is found to be inversely associated with healthcare costs^42^, ^64^ and utilization across a whole health economy^39^, ^40^, ^41^ with usage being lowest at activation Level 4.^41^, ^65^ However, multimorbid patients are known to be major users of healthcare resources.^10^, ^16^

Patient activation was found to be associated with many factors in this study. However, the only sociodemographic factor associated with lower activation was old age, in keeping with previous findings on multimorbid patients^36^ and the Australian population with comorbid diabetes and chronic kidney disease.^28^ However, mixed results were obtained in several studies on chronic populations: some found an association with age^32^, ^34^, ^39^ but others did not.^25^, ^43^ Varying relationships between other sociodemographic variables and patient activation have been reported as well. While some studies (like this one) found no relationship between activation and gender^28^, ^33^, ^43^ others suggested that women are more activated than men^66^ or vice‐versa.^25^, ^32^ Many studies have found associations between educational level and patient activation,^30^, ^33^, ^38^, ^66^ but no such association was observed in this study or in various other investigations.^34^, ^43^ These mixed study results may have been due to differences in study samples. However, previous findings have shown that PAM scores were only moderately correlated with socioeconomic status^48^ and that patients' sociodemographic characteristics (age, gender, education, and income) explained only 5%–6% of the variation in PAM scores.^67^

In this study, perceived loneliness was found to affect patient activation. More specially, feelings of loneliness and insufficient close relationships with others were more common among patients with lower patient activation. Previously, loneliness was found to be associated with low activation among military veterans with depression.^68^ These results also agree with previous findings that satisfaction with social role^38^ and social support^33^, ^36^ was positively associated with patient activation in patients with a chronic condition(s). However, living alone, or living situation, in general, was not associated with activation, in keeping with previous findings on multimorbid patients.^36^ These findings confirm the importance of taking patients' perceptions of their social relationships into account for self‐management of multimorbidity, especially when loneliness is known to be associated with multimorbidity,^13^ also in physical multimorbidity alone.^14^

In the studied population here, PAM was apparently unrelated to the number of chronic conditions, which is consistent with previous findings on adults with multimorbidity.^26^ However, perceived health, functional ability, and vitality were all significantly associated with activation: patients with lower activation scores reported worse perceived health, worse functional ability, and worse perceived vitality. Lower self‐rated health (based on diverse metrics) was also previously associated with low activation in multimorbid patients.^26^, ^33^, ^38^ Another factor significantly associated with patient activation was obesity: obese participants had lower activation than their nonobese counterparts. This is consistent with previous study findings among people with type 2 diabetes mellitus in the United States^69^ and reports showing that lower activation was associated with higher BMI in chronic patients.^25^, ^33^, ^34^ Previous studies have also stated that obesity is strongly associated with multimorbidity^7^, ^70^, ^71^ in keeping with the results obtained here: almost half of the respondents in this study fell into this category, compared to around 25% of the adult Finnish population.^51^

Patients with low and high activation also exhibited significant differences in self‐management behaviors: low activation participants ate significantly less frequently with a varied diet, complied less frequently with dietary instructions, and had lower frequencies of physical activity than those with high activation. Healthy eating^27^ and physical activity were previously found to be associated with activation.^25^, ^26^ Diet and physical activity are key lifestyle variables in the prevention and care of chronic conditions; physical activity, in particular, has been described as a polypill for several chronic diseases,^72^ has been related to increased life expectancy, and exhibits an inverse dose–response association with mortality in the multimorbid population.^73^ The results of this study also suggested that low activation in multimorbid patients predicted poorer overall adherence to care regimens. As a such, patient activation may potentially be incorporated as one tool to address the challenges of inadequate adherence, physical activity, and diets, as well as overweight and obesity, as described above.

In addition to self‐management behaviors, this study examined patients' perceptions relating to self‐management. High activated participants had more positive perceptions in terms of having energy and motivation to care for themselves, as well as feeling a sense of normality in care; meaning, for example, that they more often felt that self‐management produces well‐being and enabled them to stay healthy and was a natural part of their daily routine. Instead, low‐activation patients more frequently agreed that they did not follow recommended treatment guidelines because the guidelines did not fit their lifestyle. This is consistent with previous reports that patients with low activation may not consider their role in the care process to be important and are more likely to have low confidence in their ability to self‐manage and to feel overwhelmed as a result.^74^ The HRQoL analysis performed here reinforced the finding that such patients have significantly more difficulties with mental function and feel more depressed and distressed than high activation patients. In addition, patients with high activation felt more support for self‐management from both physicians and nurses. The relationship between activation and perceived support may be complex; perceived support may contribute to higher activation, but patients with high activation, that is, higher skills and confidence, may also find healthcare encounters more supportive. The explanation may be that they find encounters more apprehensible and moreover are more adept at getting their healthcare providers to meet their needs.^45^, ^48^ This result is supported by previous findings that a higher level of activation was associated with better patient‐professional relationships experienced by patients.^43^, ^45^ In any case, finding an association between activation and perceived support was important in itself and warrants further attention.

Because a cross‐sectional design was used, the findings demonstrate associations between patient activation and the studied factors but cannot be used to infer causality. However, it was speculated that the direction of the causal relationship between patient activation and health is likely to go in both directions, also suggested by Hibbard et al.^74^; meaning that those with low patient activation are at risk for poor self‐management and health outcomes, but also that those patients who are overwhelmed by their illness or circumstances, for example, those with poor perceived health, are likely to find self‐managing of their conditions on a day‐to‐day basis to be more difficult, and as a result, they score low patient activation. However, previous studies have shown that experiences of success in self‐management, even small, can build positive emotions and confidence and initiate positive progress.^44^ Patient activation is modifiable, and its increase can also be promoted by appropriate supportive actions. Especially, interventions tailored according to patients' activation levels have previously been shown to be effective.^48^, ^63^

### 4.1 Data quality, limitations, and strengths of the study

Inclusion criteria of the study participants were set before data collection in the study design. Multimorbidity was defined according to the definition of the Academy of Medical Sciences.^47^ Further, no attempt was made to restrict the definition of multimorbidity based on a limited list of diseases (contrary to some studies on multimorbidity^75^). Healthcare professionals recruited the study participants during healthcare encounters with patients. This may introduce some risk of bias. However, the participants were recruited from several health centers and units, and thus by many persons. Furthermore, healthcare professionals were briefed on the study and instructed to distribute questionnaires to all their patients satisfying the eligibility criteria. The limitation was that data collection was limited to one municipality. Also, the emergence of the Covid‐19 epidemic shortly after the start of data collection presented a challenge given the chosen method of data collection because it affected the functioning of healthcare organizations and reduced the number of nonurgent appointments that were held, including appointment visits for the studied chronically ill patient population. Furthermore, patients had to be completely free of respiratory infection to participate in nonurgent chronic consultations after the onset of the epidemic. It may also have reduced the willingness of patients with multimorbidity to attend appointments, as many of them belong to those at increased risk of severe Covid‐19 disease. This may partly explain the fact that, despite the rather long data collection period, the sample size remained quite small, which is a limitation of the study. However, the sample includes multimorbid patients with diverse chronic diseases and conditions. The most common conditions were hypertension, diabetes, asthma, and coronary artery diseases, all of which present important public health issues in Finland and globally. The study population was also representative of the general multimorbid population in terms of sociodemographic factors including gender and age.

To avoid measurement bias, validated instruments with appropriate answer instructions were used to collect data. To ensure the validity of PAM, all questionnaires with more than three missing responses (N/A responses were also considered missing), or those for which all questions were answered using the same response option were deleted. Cronbach *α* values were generated for all instruments and subscales; the values described good internal consistency. All the instruments used in this study relied on self‐reporting, which may introduce some risk of bias. However, this was justified, because the study focused on patients' perceptions and behaviors in their daily lives. To ensure honesty and minimize social desirability reporting bias, which is present especially in issues related to negative health behaviors, the self‐administered questionnaires were returned anonymously directly to the researcher. However, it is possible that healthier patients, and those who were more motivated for self‐management, participated more frequently. Analyses were performed carefully. The STROBE statement reporting guidelines for cross‐sectional studies were followed.^76^

### 4.2 Ethical consideration

Responsible research practices were followed according to the World Medical Association^77^ and the guidelines of the Finnish Advisory Board on Research Integrity,^78^ further complied with the European Union's General Data Protection Regulation (EU 2016/679). The study was approved by the Institutional Review Board. All instruments were used with their developers' permission, granted via a license (PAM‐13®; Insignia Health Inc.) or registration (15D)^54^ as required. All prospective participants were given detailed written information about the purpose and objectives of the study, as well as assurances regarding anonymity, confidentiality, and the voluntary nature of participation. Furthermore, the contact details of the researchers were provided so prospective participants could ask additional questions. Completing and returning the anonymous questionnaire was considered to imply informed consent for participation in the study. The data were collected, processed, and stored without identifying information. Ethical approval was thus not required.

## 5 CONCLUSION

This study on multimorbid primary healthcare patients showed that levels of activation, that is, the knowledge, skills, and confidence to manage one's own health and healthcare, in this population were rather low. Patient activation was negatively associated with old age, obesity, perceived loneliness and lack of close friends and relatives, and poor perceived health, functional ability, and vitality. However, it was not associated with other sociodemographic factors or the number of conditions suffered by the patient. The results presented here indicate that patients' perceptions of their health and functional ability as well as psychosocial factors may be important for activation and should be considered, rather than traditional socio‐demographic factors, when assessing a patient's risk of low activation. Additionally, patients with low and high activation exhibited several differences in terms of health behaviors, perceptions related to self‐management, and HRQoL. These results suggest that patient activation is important for self‐management and well‐being in multimorbid patients. Knowledge of a patient activation level may be useful when developing tailored support and interventions suited to their capabilities and needs, also considering individual needs to build more knowledge, confidence, and/or motivation. Moreover, the results highlight the importance of patient‐centredness toward a whole person in the care of patients with multimorbidity.

### 5.1 Practice implications

Patients with multimorbidity could benefit from support for patient activation to enhance self‐management needed in their everyday life. Activation support may include supporting knowledge and skills for self‐management but also strengthening confidence and understanding of the importance of the patient's own role in care. A clear determination of patient activation level in multimorbid patients could help identify patients who would benefit from greater support and what kind of support they need most to move forward. Knowledge of patient activation may help assess patients' readiness and willingness to manage their own health and facilitate adaptation of communication and care to better meet patients' abilities and needs in clinical settings such as properly delivering new information and setting realistic goals. As a such, taking patient activation into account can support person‐centered care and help to reconcile the expertise of healthcare professionals with the experience of patients in their real world, while it is important to meet a patient individually, considering their entire health and life situation. Furthermore, knowledge of a patient's activation level, for example, the proportions of low and high activation and relationships to varied self‐management behaviors, may also be useful when developing services, interventions, and care strategies for self‐management needed for this patient group; for example, activities to improve adherence.

## AUTHOR CONTRIBUTIONS

**Leila Paukkonen**: Conceptualization; data curation; formal analysis; funding acquisition; investigation; methodology; writing – original draft; and writing – review and editing. **Anne Oikarinen**: Conceptualization; methodology; supervision; and writing – review and editing. **Outi Kähkönen**: Conceptualization; methodology; and writing – review and editing. **Pirjo Kaakinen**: Conceptualization; methodology; supervision; and writing – review and editing.

## CONFLICT OF INTEREST

The authors declare no conflict of interest.

## TRANSPARENCY STATEMENT

Leila Paukkonen affirms that this manuscript is an honest, accurate, and transparent account of the study being reported; that no important aspect of the study have been omitted; and that any discrepancies from the study planned (and, if relevant, registered) have been explained.
